# Edible Plant-Derived Extracellular Vesicles for Oral mRNA Vaccine Delivery

**DOI:** 10.3390/vaccines12020200

**Published:** 2024-02-15

**Authors:** Chiara Gai, Margherita Alba Carlotta Pomatto, Maria Chiara Deregibus, Marco Dieci, Alessandro Piga, Giovanni Camussi

**Affiliations:** 1 EvoBiotech s.r.l., 10148 Torino, Italy; chiara.gai@unito.it (C.G.); margheritaalbacarlotta.pomatto@unito.it (M.A.C.P.); mdieci@evobiotech.it (M.D.); alessandro.piga@techwald.com (A.P.); 2 Department of Medical Sciences, University of Turin, 10126 Torino, Italy; mariachiara.deregibus@unito.it

**Keywords:** extracellular vesicles, exosomes, SARS-CoV-2, mRNA vaccines

## Abstract

Nucleic acid delivery through extracellular vesicles (EVs) is a well-preserved evolutionary mechanism in all life kingdoms including eukaryotes, prokaryotes, and plants. EVs naturally allow horizontal transfer of native as well as exogenous functional mRNAs, which once incorporated in EVs are protected from enzymatic degradation. This observation has prompted researchers to investigate whether EVs from different sources, including plants, could be used for vaccine delivery. Several studies using human or bacterial EVs expressing mRNA or recombinant SARS-CoV-2 proteins showed induction of a humoral and cell mediated immune response. Moreover, EV-based vaccines presenting the natural configuration of viral antigens have demonstrated advantages in conferring long-lasting immunization and lower toxicity than synthetic nanoparticles. Edible plant-derived EVs were shown to be an alternative to human EVs for vaccine delivery, especially via oral administration. EVs obtained from orange juice (oEVs) loaded with SARS-CoV-2 mRNAs protected their cargo from enzymatic degradation, were stable at room temperature for one year, and were able to trigger a SARS-CoV-2 immune response in mice. Lyophilized oEVs containing the S1 mRNA administered to rats via gavage induced a specific humoral immune response with generation of blocking antibodies, including IgA and Th1 lymphocyte activation. In conclusion, mRNA-containing oEVs could be used for developing new oral vaccines due to optimal mucosal absorption, resistance to stress conditions, and ability to stimulate a humoral and cellular immune response.

## 1. Introduction

Vesiculation is a mechanism of cell-to-cell communication present in all life kingdoms [1]. Vesicles actively secreted from cells were originally studied in eukaryotes and were subsequently found to be present in prokaryotes and plants. Secreted vesicles are constituted by a bilayer lipid membrane derived from the cell of origin and may carry molecular messages including bioactive lipids, proteins, and nucleic acids that can be shared from the cell of origin to neighboring or distant cells [2,3]. Membrane vesicles containing cytosolic components included in the lipid bilayer were suggested to be inclusively named extracellular vesicles (EVs), comprising exosomes secreted from the multivesicular bodies and microvesicles secreted by budding of the plasma membrane [2,3]. Originally, plasma membrane-derived vesicles were named microvesicles, a misleading term as it included vesicles originated by membrane buddings of a wide range of sizes, comprising pre-apoptotic larger vesicles (500–1000 nm) and smaller vesicles in the nano range (50–250 nm) that are actively released by healthy cells. It is therefore preferred to refer to the latter as ectosomes [4]. The subset of small vesicles indicated as exosomes (30–120 nm) originates from inside the cell membrane in three subsequent phases: internal membrane budding of the plasma membrane with the first formation of early and late endosomes that merge into multivesicular bodies, the fusion of the multivesicular body membrane with the plasma membrane, and the subsequent release of nanovesicles in the extracellular space as exosomes [5,6]. During early exosome formation, proteins and nucleic acids can be captured and loaded into late exosomes that originate the multivesicular bodies and finally generate exosomes [7].

The molecular mechanisms involved in ectosome and exosome biogenesis are partly similar and partly distinctive. However, the mechanism of assembling and sorting of secreted vesicles may differ in different cell types, and a common mechanism for all cells has not yet been identified [8]. The formation of multivesicular bodies has been linked to the involvement of the endosomal sorting complex required for transport (ESCRT), the apoptosis gene 2-interacting protein X (ALIX), and tetraspanins (CD63, CD81, and CD9). The intracellular transport of vesicles to the cell surface involves Ras-associated binding protein (RAB) and several proteins of the cytoskeleton. Exosome exocytosis occurs after interaction with the N-ethylmaleimide-sensitive fusion protein attachment protein receptor (SNARE) protein [9] and involves the activation of the cytoskeleton, regulated by p53 protein [10]. However, an ESCRT-independent exosome formation has also been described [11]. Ectosome formation by plasma membrane budding is related to calcium influx, calpain, and cytoskeleton reorganization [2,3,4]. The formation of microvesicles/ectosomes depends on two physical mechanisms described by Schara et al. [12]: curvature due to lateral redistribution of membrane components generating membrane nanodomains, and the attractive forces between membranes. The asymmetric phospholipid distribution of plasma membranes is modulated by the intracellular level of calcium and by enzymes known as flippase, floppase, and scramblase [13]. Translocation of phosphatidylserine from the inner to the outer leaflet of the plasma membrane due to translocase inhibition caused by calcium ions influx and scramblase activation exposes large amounts of phosphatidylserine and lipid rafts-associated proteins [14]. The ensuing reorganization of the cytoskeleton allows the detachment of plasma membrane projections from cortical actin. Moreover, calcium influx favors calpain-dependent cleavage of talin, activin, and gelsolin, which in turn cleave actin-capping proteins [15].

During biogenesis, several biologically active molecules are recruited within EVs, either through being constituents of the plasma membrane such as membrane receptors and bioactive lipids, by interaction with membrane components, or through being constituents of the cytosol, which remain included within the EV membrane. They may include metabolites, cytokines, and nucleic acids [16,17].

Several studies have shed light on the physio-pathological role of secreted vesicles abundantly present in the human body [18,19]. The role of EVs as a mechanism of cell-to-cell communication has been ascribed to their ability to transfer modulatory transcripts from neighboring or distant cells. In particular, the role of EV-mediated nucleic acid transfer in the induction of functional and phenotypic changes in recipient cells has been demonstrated. Ratajczak et al. [20] first showed that vesicles secreted from embryonic stem cells may induce epigenetic changes in human hemopoietic stem cells. A vesicle-mediated horizontal transfer of mRNA from endothelial progenitors was shown to activate an angiogenic program in quiescent endothelial cells [21]. Valadi et al. [22] demonstrated that EVs may transfer not only biologically active mRNA, but also microRNA. Subsequent studies investigated different subsets of nucleic acids incorporated in EVs of different origin [23,24,25,26,27,28]. It was found that EVs allow horizontal transfer of native but also exogenous functional nucleic acids. The first demonstration of the fact that an exogenous mRNA could be loaded into EVs, transferred to target cells, and translated into proteins was shown using green fluorescent protein (GFP) mRNA [21,29]. Therefore, EVs could be useful carriers for drug delivery, and they may overcome the limitations of synthetic drug carriers, including polymeric nanoparticles and liposomes that have found wide applications in clinical settings [30,31]. EVs have emerged as an appealing delivery system compared to synthetic carriers due to their biosafety and intrinsic abilities to cross biological barriers and reach targets [32,33]. These natural carriers appear particularly suitable for the delivery of nucleic acids because the protective bilayer membrane prevents enzyme degradation and confers stability. Moreover, their low immunogenicity may allow repeated administrations [34].

The interest in EVs as candidates for the development of new vaccine strategies relies on their ability to carry different molecules at defined anatomical sites [32,35], allowing antigen presentation and activation of an immune response [36,37].

## 2. EV Loading Methods

To optimize drug delivery, it is necessary to modify EV cargo or surface molecules [38]. Several studies have investigated various methods of cargo modification to exploit EVs as a drug delivery system [39,40,41]. The two main strategies are the indirect modification of the EV cargo via manipulation of the donor cells, and direct interventions on purified EVs. The first strategy is based on the loading of donor cells with a specific molecule, or the induction of genetic modification to obtain EVs selectively enriched with the desired molecules [36,42]. Genetic modification can be induced using expression vectors for selected genes fused with EV native surface proteins. This enables the directed secretion of EVs expressing the target peptide. This strategy can be used to modify EVs for drug or gene delivery purposes. However, vector selection should consider the potential risks of immunogenicity, teratogenesis, and pathogenicity when devised for clinical application. Moreover, this approach for loading functional mRNA met substantial failure, as despite the presence of mRNA within EVs using a bioluminescent reporter, the effect was caused by plasmids loaded into EVs [43].

The direct modification of purified EVs can be either an active or a passive process [38]. Passive loading is based on the incubation of EVs with a high concentration of a defined molecule, allowing diffusion through the EV membrane. The efficiency of this process may depend on hydrophobicity and/or the charge of loading molecules, as well as the time of incubation [44]. One of the merits of passive loading is that it does not damage the EV structure. EVs contain several RNA binding proteins that may be instrumental in endogenous RNA binding [21,45]. Specific proteins able to bind RNAs present on the surface of EVs can enable EV loading [46,47]. Annexin A2 was identified as one of the surface molecules able to bind exogenous RNAs in human serum EVs, allowing loading of functional miRNAs [48]. siRNAs conjugated with cholesterol, as well as lipophilic drugs, also allow successful passive loading into EVs [49,50]. To enhance loading efficacy, several active loading techniques have also been developed based on alterations of EV membrane permeability. These include electroporation, osmotic shock, sonication, and the use of tension-active molecules [51,52]. Several studies have shown successful EV loading via electroporation for siRNA [53], miRNA [54], and pDNA [55], even if with low efficiency. However, electroporation, despite being effective, may induce aggregation of RNA molecules and damage to the EV membrane and surface [56].

The membrane anchor technique has shown better efficiency for siRNA loading and is largely dependent on siRNA/EV ratio [49]. For siRNA and miRNA, loading has also successfully been performed using cationic transfection [49,57,58]. These techniques have a variable efficiency of loading depending on the source of vesicles and on the type of loading molecules. Further limitations of these techniques are related to the stability of different EVs and their resistance to membrane disruption. Most of the direct loading techniques are based on enhancement of EV membrane permeability and are associated with a differential out/in gradient of loading molecules. To our knowledge, no formal study has been performed on native content depletion after the loading of exogenous molecules, and the magnitude of this effect may vary depending on the type of techniques used. In experiments performed on human EVs engineered by electroporation [59] we observed the depletion of selected endogenous miRNAs used as markers after loading.

Several techniques have also been developed for EV surface functionalization in order to modify their biodistribution and to achieve targeted drug delivery. These techniques are based on surface engineering using genetic or chemical modification or the generation of hybrid membranes [32]. Generation of EV-liposome hybrids exploiting the spontaneous ability of EV plasma membranes to fuse with lipid nanoparticles has been used to deliver large molecules [60], including CRISPR–Cas9 for gene editing [61].

## 3. Plant-Derived EVs

As EVs of human origin exhibit several technical difficulties relating to manufacturing scalability and are extremely high cost, especially when isolated for drug delivery purposes, plant-derived EVs are emerging as an attractive alternative solution.

**Figure 1:**
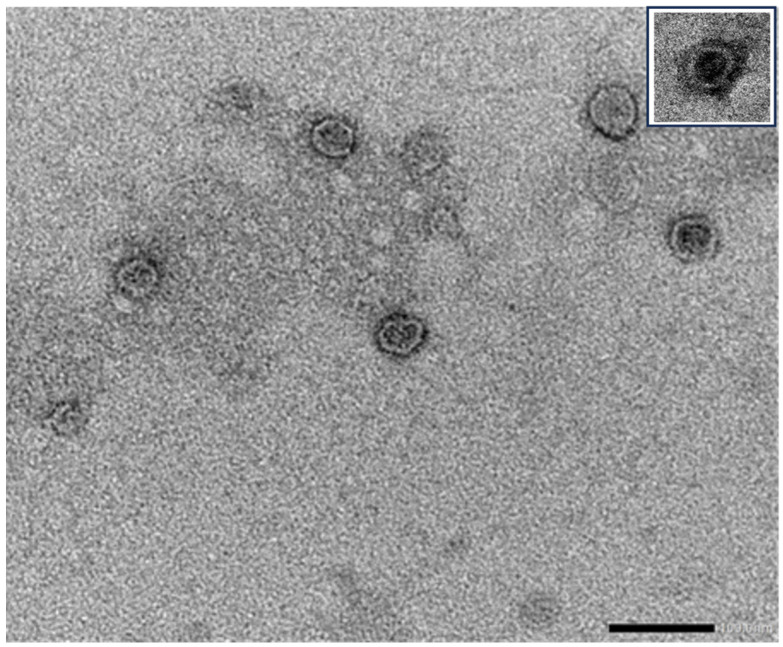
Transmission electron microscopy of plant EVs. Representative image of EVs purified from orange juice negatively stained with NanoVan (Nanoprobes Inc., Yaphank, NY, USA) and examined with a Jeol JEM 1400 Flash transmission electron microscope (Jeol, Peabody, MA, USA) (bar 100 nm)**.** The inset shows a representative image of an EV purified from cultured media of human stem cells, stained and observed using the same procedures [64].

Several studies have demonstrated that plants are able to secrete EVs morphologically similar to those released by eukaryotic cells (for review see [62,63]). Transmission electron microscopy has revealed that plant-derived EVs have a spherical appearance with a bilayer membrane and an electron-dense core, similar to human EVs (Figure 1).

The presence of vesicles morphologically resembling exosomes released from multivesicular bodies of a plant culture was originally described by Halperin and Jensen [65]. Subsequent studies demonstrated the origin of EVs from multivesicular bodies after fusion with the cell plasma membrane, similar to as described in eukaryotes [66]. As in eukaryotes, the ESCRT machinery is considered to be relevant for multivesicular bodies-dependent formation of EVs, since ESCRT I, II, and III, but not ESCRT-0, and accessory proteins are conserved in plants [67]. Several candidate molecules were proposed to substitute ESCRT-0, which is involved in ubiquitinated cargo and recruitment of ESCRT I, II, and III complexes, such as the FYVE domain protein required for endosomal sorting and the orthologue of mammalian TOM-1 [65]. Similarities between plant EV biogenesis and that of mammalian EVs with respect to the involvement of ESCRT genes have been recently suggested following the detection of TET8 and TET9 tetraspanins and PEN1 syntaxin protein in plant EVs [68].

Exocyst-positive organelle (EXPO)-mediated secretion, autophagosome-mediated secretion, and vacuole–PM fusion have also been described as pathways for biogenesis alternative to multivesicular bodies-dependent plant EV secretion [69,70].

The mechanisms involved in the cell wall crossing of EVs are unclear. It has been suggested that some hydrolases associated with EVs, as well as their lipidic structure, may favor transition through the cell wall pores [71,72].

Several studies focused on plant EV structure and cargo have revealed their potential physiological role in the plant response to pathogens, in interactions with microbes, and in the organization of cell walls [73,74,75].

Edible plants are an abundant natural source of EVs, and they easily allow EV extraction with high yields on a large scale. In addition, EVs derived from edible plants are ideal for the oral administration of drugs and nucleic acids because they are nontoxic and non-immunogenic due to oral tolerance [76] in most of the human population.

As recently observed, EVs are contained in food, and they physiologically interact with human metabolism. After intestinal absorption, food-derived EVs transfer molecules modulating several metabolic pathways [77]. For instance, it has been shown that edible plant-derived EVs induce the expression of anti-inflammatory cytokine genes and antioxidant molecules that maintain intestinal homeostasis [78]. Plant-derived EVs have been shown to have natural beneficial effects for human health and potential therapeutic activities such as antitumor, anti-inflammatory, and wound healing properties, while no adverse effects have been reported [78]. The unique lipidic composition of plant-derived EV membranes confers high resistance to physical and chemical stresses. This makes them particularly suitable for engineering and drug loading for delivery purposes [79].

Several studies in eukaryotes have shown that nucleic acids incorporated in EVs are protected from degrading enzymes present in all biological fluids [20,21,22,23,24,25,26,27,28]. This property has been exploited to cultivate the use of plant-derived EVs as a delivery system for nucleic acids. For instance, it has been shown that EVs derived from grapefruit loaded with miR-17 inhibited the progression of brain tumors in mice [80]. Similarly, ginger-derived EVs loaded with siRNA showed a beneficial effect in the treatment of ulcerative colitis [81]. Several new strategies for engineering plant-derived EVs have been recently developed, allowing not only the incorporation of small RNAs but also mRNA and exogenous DNA plasmids [82,83,84], suggesting that plant EVs are adaptable to nucleic acids of a wide range of sizes.

## 4. EVs as a Delivery System for Vaccines

It has been shown that EVs may trigger cell and humoral immune responses by carrying antigens on their surface, along with immunostimulatory molecules [85,86]. For instance, EVs containing tumor-derived antigens can interact with antigen-presenting cells, triggering a CD8+ T cell-mediated immune response in mice vaccinated with tumor-EVs [87]. Compared to soluble molecules, the antigens associated with EVs released after DNA vaccination were shown to be more immunogenic in mice [88]. Based on these properties, EVs have been investigated in clinical trials of tumor immunotherapy [89].

The potential use of bacteria outer membrane vesicles to develop vaccines has also become a topic of interest [90]. Bacteria also secrete EVs, which play a crucial role in cross-talk with the host, playing a relevant role in pathogenesis [91]. EVs secreted by H. pylori carry several bacterial constituents acting as pathogenic factors for the host [92]. Since bacterial EVs carry antigenic components of bacteria, they may be exploited to stimulate the host immunity. By interacting with dendritic cells, they may activate both innate and adaptive immune responses [90]. Since bacterial EVs are non-replicative, they may represent a suitable strategy to immunize the host without the risk of infection associated with intact bacterial cells. It has been shown that intranasal immunization with EVs derived from Neisseria meningitidis induces an effective mucosal immune response with the production of specific IgG and IgA [92]. Moreover, it has been shown that EV-based vaccines trigger not only the humoral but also the cell-mediated immune response [93]. This represents an advantage in the targeting of mucosal tissues as compared to existing adjuvants.

Genetically modified bacteria may be used to generate EVs displaying neoantigens, acting both as an adjuvant and immunogen for vaccine development [90,94]. The adjuvant activity of bacterial EVs has been associated with the presence of EV-associated LPS. LPS toxicity may represent a limitation, and a balance should be found in the production of EVs with low LPS toxicity before their potential clinical use. Another limitation is the low yield of EV production from bacteria, and several studies are currently ongoing to define the optimal stress and temperature culture conditions for EV secretion.

In the viral vaccine field, a growing interest in EVs was stimulated by the observation that virally infected cells released EVs carrying viral antigens able to trigger an immune response [95,96]. In COVID-19 patients, the SARS-CoV-2 Spike (S) protein presented on EVs was correlated with the severity of the disease [97]. Moreover, S protein-associated EVs detectable in infected patients were suggested to be instrumental in triggering a humoral-specific immune response [97,98,99]. Therefore, the possible use of EVs as carriers for viral antigens has been investigated to develop vaccines, as summarized in Table 1.

A SARS-CoV-2 vaccine was developed based on EVs from Salmonella typhimurium decorated with Spike receptor-binding domain (RBD). Golden Syrian hamsters (Mesocricetus auratus) immunized intranasally developed high titers of blood anti-Spike RBD IgG as well as a mucosal response, and after infection with the virus they developed much less severe lung pathology [105]. Genetically engineered dendritic cells expressing SARS-CoV-2 S protein were used to generate extracellular blebs that were used as vaccines, inducing the production of neutralizing antibodies [106]. Wang et al. [107] developed a vaccine using human lung-derived EVs conjugated with recombinant SARS-CoV-2 RBD. The vaccine efficiently immunized mice and induced variance in liposomes, triggering specific IgG, humoral IgA, and CD4+ and CD8+ T cell responses. Immunized hamsters encountered a significant reduction in severe pneumonitis after infection with live SARS-CoV-2. Interestingly, the EV-based vaccine was demonstrated to be stable for three months after lyophilization.

Popowski et al. [111] developed an inhalable vaccine in the form of a dry powder containing lung-derived EVs carrying an mRNA encoding for SARS-CoV-2 S protein. Administration of the vaccine in vivo was shown to elicit IgG and IgA immune responses at a significantly higher rate than liposomes. The comparison of EVs with synthetic liposomes showed superior efficacy of EVs due to a better distribution into bronchioles and lung parenchyma after nebulization, both in rodents and in non-human primates [108,111]. In addition, dry mRNA-loaded EVs remained functional when stored at room temperature for one month. Tsai et al. [112] created a vaccine based on HEK293-derived EVs fused with lipid-coated mRNAs encoding SARS-CoV-2 S and N proteins. Unlike RNA-loaded synthetic lipid nanoparticles, which possess marked cell toxicity, the EV vaccine was devoid of any toxicity both in vitro and in vivo and induced a long-lasting humoral and cellular immune response.

Taken together, these findings confirm that EVs are a good candidate for the development of innovative, versatile, and effective vaccine formulations.

## 5. Edible Plant-Derived EVs as a Platform for Mucosal Vaccine Delivery

The large-scale production of EVs from human cells is still problematic, due to the extremely expensive and time-consuming manufacturing process. To circumvent these difficulties, EVs derived from transfected yeast or bacteria have been proposed as an alternative [105]. However, the use of genetically modified organisms (GMOs) as a source of EVs may pose questions relative to the complex and diverse legislation regulating GMOs in different countries.

Edible plants may represent a largely available and low-cost source for the large-scale production of EVs. EVs are particularly abundant in the juice of some edible plants and can be easily extracted with scalable techniques. Moreover, edible plant EVs are commonly ingested as part of fruits and vegetables; thus, they are nontoxic and nonimmunogenic. This makes them a good candidate for oral drug delivery. Numerous studies have investigated the loading techniques of plant-derived EVs with nucleic acids and have shown that EVs can protect loaded nucleic acids from enzymatic degradation and deliver them in an intact and functional form [113,114]. Techniques used for plant EV loading include electroporation, which causes transient pore formation and allows nucleic acid entrance into EVs; sonication of EVs in the presence of nucleic acids to alter membrane structures; passive nucleic acid internalization in the presence of appropriate salt, pH, and temperature conditions; and mechanically (extrusion techniques) or chemically increased membrane permeability to allow nucleic acids entrance. The efficacy of these techniques varies depending on the EV source and the nucleic acid type [113].

**Figure 2:**
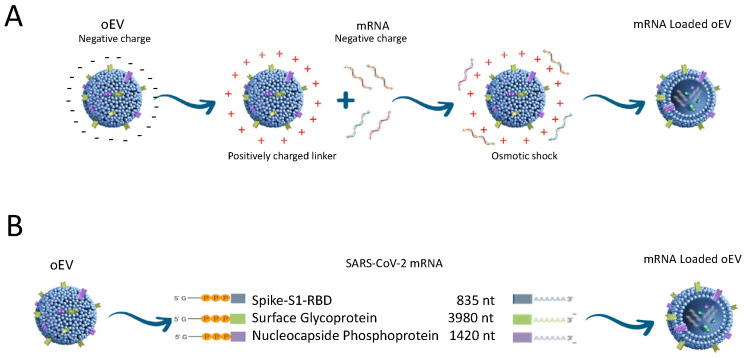
Schematic representation of oEV loading procedure. Panel (**A**): oEV engineering based on charge interactions and controlled osmotic stress. Panel (**B**): oEVs were efficiently loaded with mRNAs coding SARS-CoV-2 antigens.

Using a proprietary technique [115], we engineered EVs purified from orange juice (Citrus sinensis) (oEVs) with SARS-CoV-2 mRNA coding for Spike S1 subunit (S), Full Spike (FS), and nucleocapsid (N) proteins using cation-based interaction combined with controlled osmotic shock (Figure 2) [110].

The efficiency of loading was about 72 ± 11% for all the studied mRNAs, with a loading capacity of 3.51 ± 1.09 ng/10^11^ oEVs independently from the mRNA length (S1 mRNA 669 nt; N mRNA 1260 nt and FS mRNA 3822 nt). Once incorporated in oEVs, mRNA was protected from RNase and gastroenteric enzyme degradation. Protection was due to encapsulation into oEVs, since Triton X-100 permeabilization of oEV lipid membrane abrogated the resistance to RNase. In vitro experiments demonstrated that the oEV-mediated delivery of viral mRNAs to macrophages was followed by translation into N, S1, and FS proteins and lymphocyte activation [110]. Moreover, oEV incorporation of mRNA conferred resistance at room temperature up to one year after lyophilization [110]. S1 or FS-loaded oEVs in a liquid formulation without adjuvants were administered orally via gavage in vivo to mice models and were compared with intra-muscle administration. The vaccination with S1 or FS-loaded oEVs induced comparable production, as related to both the oral and intra-muscular administrations, of specific IgM and IgG and of neutralizing antibodies. Interestingly, the oral administration route induced a significant production of specific secretory IgA. IgA antibodies are the first mucosal barrier in adaptive immunity [116] and represent one of the major advantages of oral vaccines [117,118]. Moreover, mice immunized with S1-loaded oEVs showed specific splenic lymphocyte activation after stimulation with the S1 peptide with a Th1 profile of cytokine secretion. This observation agrees with studies that showed prevalent activation of the Th1 response after vaccination with mRNA coding for SARS-CoV-2 antigens [119]. Similarly, Zhang et al. [120] used a vaccine based on nanoparticle-encapsulated mRNA encoding the SARS-CoV-2 RBD and showed that, in vitro, stimulated splenocytes induced a Th1 activation with significant secretion of IFN-γ and IL-2.

Biodistribution studies of orally administered oEVs showed that the vaccine reaches the small intestine, where the majority of immune cells are localized, but most of the oEVs were absorbed at the gastric level [109]. To avoid gastric dispersion, a formulation of lyophilized S1-loaded oEVs encapsulated in gastro-resistant capsules was prepared [110]. Capsules were used for immunizing rats and were administered orally via gavage. As previously observed in mice, rats developed a humoral immune response, involving the production of blocking antibodies and specific IgM, IgG, and IgA. The vaccination also triggered a Th1 immune response [110]. Moreover, the stability of the vaccine formulation was evaluated after one year and showed intact and functional mRNA inside lyophilized oEVs [110].

Experience relating to mRNA-based oral vaccines is so far very limited and research often stalls due to mRNA fragility and the need for formulations that provide in vivo stability [116]. Mucosal vaccines currently undergoing clinical trials are mainly based on protein antigens and live attenuated viruses and are preferentially delivered via viral vectors [117]. The formulation of lyophilized mRNA-EVs could be an efficient strategy for oral vaccine development due to the fact that they are stable at room temperature, optimally absorbed at the mucosal level, and able to induce an immune response [110,120].

In general, oral vaccine administration has several pros and cons (reviewed in [37,118,121]). Oral vaccines have several advantages, including improved patient compliance, the possibility of self-administration, no needle-associated risks, logistic advantages relating to storage and distribution, and induction not only of IgG but also of IgA and T cell immune responses. More than ninety per cent of pathogens enter the body trough gastrointestinal, respiratory, and urinary mucosae. Therefore, the mucosal immune response is particularly relevant for preventing pathogen invasion. Indeed, vaccination through the mucosal route elicits production of IgA, which represents the first mucosal barrier in the adaptive immune response. The oral administered vaccines require much less purification than the injectable ones due to the non-sterile environment for the abundant microbiome of gastrointestinal tract. This may allow a significant reduction in manufacturing costs. The main challenges are related to vaccine degradation due to the gastroenteric pH, and the presence of proteolytic enzymes and bile salt. However, these obstacles may, at least in part, be overcome by formulation with gastro-resistant capsules. Moreover, absorption of the vaccine at mucosal levels usually requires the administration of high and repeated doses of antigens, and that might induce oral tolerance. The development of oral vaccines is also impaired by inefficient absorption and scarceness of mucosal adjuvants. Therefore, research is focusing on carriers that may implement mucosal absorption such as nanoparticles that encapsulating the vaccine may protect and stabilize it (reviewed in [122]).

The use of intranasal administration constitutes a possible approach for the induction of mucosal immunity in order to reduce the risk of inactivation due to gastroenteric enzymes, and several clinical trials are currently ongoing [116,117].

The intranasal application of EV-based SARS-CoV-2 mRNA vaccines has been shown to induce responses in resident memory T cells and B cells, as well as stimulating IgA production [105,106,107,108,111,112].

We performed intranasal administration of oEVs loaded with S1 mRNA in mice, showing the induction of a humoral and T cell immune response as obtained with oral immunization [109]. In this study, we demonstrated the presence of specific IgA in the bronchoalveolar lavage of the immunized mice. We used a nasal drop of S1-loaded oEVs in solution, but lyophilized oEVs can also be administered via direct nebulization. The efficacy of intra-nasal immunization using salmonella typhimurium EVs [105] or lung-derived EVs [107,108,111] as carriers for mRNA coding SARS-CoV-2 antigens has been previously shown.

## 6. Conclusions

In conclusion, EVs as carriers of bacterial/viral antigens are good candidates for the development of new vaccine strategies. Engineered EVs derived from yeast, bacteria, or mammalians may also allow effective delivery of mRNA vaccines. However, these sources of EVs have limitations related to low yield and complex and expensive production and purification techniques.

Most of the preclinical studies for vaccine delivery used human derived EVs purified from cell culture conditioned media. Nevertheless, the requirement for the stringent use of clean rooms under good manufacturing practice (GMP) conditions during the entire cell culture process, along with the required use of stringent sterile purification standards, are the major impediments to their use in clinical applications due to high cost and low productivity. Thus, for potential industrial use, the focus was turned to plant-derived EVs that due to already being present in nature are an extractive product and do not require cell culture [78,79,80,81]. Moreover, an additional cost reduction can be achieved through using the EVs purified from the juice of edible plants, such as orange juice, where EVs are particularly abundant. Using filtration methods such as tangential flow filtration to avoid ultracentrifugation, purification of EVs can be achieved at a high degree of productivity. EVs from oranges can be considered part of a circular green economy due to the possibility of reusing fibers and peels discarded during purification, as well as the use of the juice, once deprived of EVs, for other commercial purposes.

Edible plant-derived EVs have the advantage of enabling oral administration, which elicits mucosal immunity and provides a first line of defense at the site of virus entry. Edible plant EVs are effective delivery systems because they can protect nucleic acids from enzyme degradation and environmental stress conditions. The native membrane envelope facilitates entry into target cells and the delivery of cargo. Compared to other synthetic delivery systems (e.g., LNP, synthetic lipoparticles, and adenovirus), plant-derived EVs have several advantages. They are biocompatible and do not elicit cytotoxicity. Being a natural product and part of the diet, they have an optimal safety profile. Moreover, their high resistance to stress allows lyophilization and storage at room temperature. For all these reasons, plant-derived EVs can be further studied as a versatile system for the mucosal delivery of mRNA vaccines.

## Figures and Tables

**Table 1:** List of studies using EVs as a delivery system for vaccine delivery.

Vaccine Type	Administration Route	Serum Antibodies	Presence of Neutralizing Antibodies	IFNγ Secretion	References
SARS-S protein in EVs (compared to AAV)	Footpad injection	Presence of serum antibodies	Yes	/	[100]
SARS-CoV-2 S or N protein on the surface of EVs	i.m.	Presence of serum antibodies	Yes	Yes	[101,102]
Endogenous engineered EVs expressing SARS-CoV-2 antigens	i.m.	Presence of serum antibodies	/	Yes	[103,104]
EVs of Salmonella typhimurium decorated with SARS-CoV-2 S protein	i.n.	Presence of serum antibodies: IgG, IgM, IgA	Yes	/	[105]
EVs from engineered DCs	s.c.	Presence of serum antibodies: IgG	/	/	[106]
SARS-CoV-2 S mRNA in Lung-derived EVs (compared to LNPs)	i.n.	Presence of serum antibodies IgG, IgA	/	/	[107]
SARS-CoV-2 S and N mRNA in EVs (compared to LNPs)	i.m.	Presence of serum antibodies	/	/	[108]
SARS-CoV-2 S mRNA in oEV, liquid	i.m., i.n., oral	Presence of serum antibodies: IgG, IgM, IgA	Yes	Yes	[109]
SARS-CoV-2 S mRNA in oEV, lyophilized	oral	Presence of serum antibodies: IgG, IgM, IgA	Yes	Yes	[110]

Abbreviations: S: spike protein, N: nucleocapsid protein, i.m.: intramuscular, i.n.: intranasal, s.c.: subcutaneous.

## References

[1] Ratajczak J., Wysoczynski M., Hayek F., Janowska-Wieczorek A., Ratajczak M.Z. (2006). Membrane-derived microvesicles: Important and underappreciated mediators of cell-to-cell communication. Leukemia.

[2] Raposo G., Stoorvogel W. (2013). Extracellular vesicles: Exosomes, microvesicles, and friends. J. Cell Biol..

[3] Camussi G., Deregibus M.C., Bruno S., Cantaluppi V., Biancone L. (2010). Exosomes/microvesicles as a mechanism of cell-to-cell communication. Kidney Int..

[4] Cocucci E., Racchetti G., Meldolesi J. (2008). Shedding microvesicles: Artefacts no more. Trends Cell Biol..

[5] Ha D., Yang N., Nadithe V. (2016). Exosomes as therapeutic drug carriers and delivery vehicles across biological membranes: Current perspectives and future challenges. Acta Pharm. Sin. B.

[6] Fu S., Wang Y., Xia X., Zheng J.C. (2020). Exosome engineering: Current progress in cargo loading and targeted delivery. NanoImpact.

[7] Chen H., Wang L., Zeng X., Schwarz H., Nanda H.S., Peng X., Zhou Y. (2021). Exosomes, a new star for targeted delivery. Front. Cell Dev. Biol..

[8] Johnstone R.M. (2006). Exosomes biological significance: A concise review. Blood Cells Mol. Dis..

[9] Colombo M., Raposo G., Thery C. (2014). Biogenesis, secretion, and intercellular interactions of exosomes and other extracellular vesicles. Annu. Rev. Cell Dev. Biol..

[10] Yu X., Harris S.L., Levine A.J. (2006). The regulation of exosome secretion: A novel function of the p53 protein. Cancer Res..

[11] Wei D., Zhan W., Gao Y., Huang L., Gong R., Wang W., Zhang R., Wu Y., Gao S., Kang T. (2021). RAB31 marks and controls an ESCRT-independent exosome pathway. Cell Res..

[12] Schara K., Jansa V., Sustar V., Dolinar D., Pavlic J.I., Lokar M., Kralj-Iglic V., Veranic P., Iglic A. (2009). Mechanisms for the formation of membranous nanostructures in cell-to-cell communication. Cell Mol. Biol. Lett..

[13] Hugel B., Martinez M.C., Kunzelmann C., Freyssinet J.M. (2005). Membrane microparticles: Two sides of the coin. Physiology.

[14] Del Conde I., Shrimpton C.N., Thiagarajan P., López J.A. (2005). Tissue-factor-bearing microvesicles arise from lipids rafts and fuse with activated platelets to initiate coagulation. Blood.

[15] Pap E., Pallinger E., Pasztoi M., Falus A. (2009). Highlights of a new type of intercellular communication: Microvesicle-based information transfer. Inflamm. Res..

[16] de Abreu R.C., Fernandes H., da Costa Martins P.A., Sahoo S., Emanueli C., Ferreira L. (2020). Native and bioengineered extracellular vesicles for cardiovascular therapeutics. Nat. Rev. Cardiol..

[17] Zhang Y., Liu Y., Liu H., Tang W.H. (2019). Exosomes: Biogenesis, biologic function and clinical potential. Cell Biosci..

[18] Gurung S., Perocheau D., Touramanidou L., Baruteau J. (2021). The exosome journey: From biogenesis to uptake and intracellular signalling. Cell Commun. Signal.

[19] Doyle L.M., Wang M.Z. (2019). Overview of extracellular vesicles, their origin, composition, purpose, and methods for exosome isolation and analysis. Cells.

[20] Ratajczak J., Miekus K., Kucia M., Zhang J., Reca R., Dvorak P., Ratajczak M.Z. (2006). Embryonic stem cell-derived microvesicles reprogram hematopoietic progenitors: Evidence for horizontal transfer of mRNA and protein delivery. Leukemia.

[21] Deregibus M.C., Cantaluppi V., Calogero R., Lo Iacono M., Tetta C., Biancone L., Bruno S., Bussolati B., Camussi G. (2007). Endothelial progenitor cell derived microvesicles activate an angiogenic program in endothelial cells by a horizontal transfer of mRNA. Blood.

[22] Valadi H., Ekström K., Bossios A., Sjöstrand M., Lee J.J., Lötvall J.O. (2007). Exosome-mediated transfer of mRNAs and microRNAs is a novel mechanism of genetic exchange between cells. Nat. Cell Biol..

[23] Guduric-Fuchs J., O’Connor A., Camp B., O’Neill C.L., Medina R.J., Simpson D.A. (2012). Selective extracellular vesicle-mediated export of an overlapping set of microRNAs from multiple cell types. BMC Genom..

[24] Ji H., Chen M., Greening D.W., Rai A., Zhang W., Simpson R.J. (2014). Deep sequencing of RNA from three different extracellular vesicle (EV) subtypes released from the human LIM1863 colon cancer cell line uncovers distinct miRNA-enrichment signatures. PLoS ONE.

[25] Nolte’t Hoen E.N., Buermans H.P., Waasdorp M., Stoorvogel W., Wauben M.H., ‘t Hoen P.A. (2012). Deep sequencing of RNA from immune cell-derived vesicles uncovers the selective incorporation of small non-coding RNA biotypes with potential regulatory functions. Nucleic Acids Res..

[26] Cai J., Wu G., Jose P.A., Zeng C. (2016). Functional transferred DNA within extracellular vesicles. Exp. Cell Res..

[27] Lázaro-Ibáñez E., Sanz-Garcia A., Visakorpi T., Escobedo-Lucea C., Siljander P., Ayuso-Sacido A., Yliperttula M. (2014). Different gDNA content in the subpopulations of prostate cancer extracellular vesicles: Apoptotic bodies, microvesicles, and exosomes. Prostate.

[28] Fischer S., Cornils K., Speiseder T., Badbaran A., Reimer R., Indenbirken D., Grundhoff A., Brunswig-Spickenheier B., Alawi M., Lange C. (2016). Indication of horizontal DNA gene transfer by extracellular vesicles. PLoS ONE.

[29] Aliotta J.M., Pereira M., Johnson K.W., de Paz N., Dooner M.S., Puente N., Ayala C., Brilliant K., Berz D., Lee D. (2010). Microvesicle entry into marrow cells mediates tissue-specific changes in mRNA by direct delivery of mRNA and induction of transcription. Exp. Hematol..

[30] Butreddy A., Kommineni N., Dudhipala N. (2021). Exosomes as naturally occurring vehicles for delivery of biopharmaceuticals: Insights from drug delivery to clinical perspectives. Nanomaterials.

[31] Elsharkasy O.M., Nordin J.Z., Hagey D.W., de Jong O.G., Schiffelers R.M., Andaloussi S.E., Vader P. (2020). Extracellular vesicles as drug delivery systems: Why and how?. Adv. Drug Deliv. Rev..

[32] Weng Z., Zhang B., Wu C., Yu F., Han B., Li B., Li L. (2021). Therapeutic roles of mesenchymal stem cell-derived extracellular vesicles in cancer. J. Hematol. Oncol..

[33] Modani S., Tomar D., Tangirala S., Sriram A., Mehra N.K., Kumar R., Khatri D.K., Singh P.K. (2021). An updated review on exosomes: Biosynthesis to clinical applications. J. Drug Target..

[34] Einabadi M., Ai J., Kargar M., Kafilzadeh F., Taghdiri Nooshabadi V., Jamali H. (2020). Mesenchymal cell-derived exosomes as novel useful candidates for drug delivery. Arch. Neurosci..

[35] Santos P., Almeida F. (2021). Exosome-based vaccines: History, current state, and clinical trials. Front Immunol..

[36] Kučuk N., Primožič M., Knez Ž., Leitgeb M. (2021). Exosomes engineering and their roles as therapy delivery tools, therapeutic targets, and biomarkers. Int. J. Mol. Sci..

[37] Montaner-Tarbes S., Fraile L., Montoya M., Del Portillo H. (2021). Exosome-based vaccines: Pros and cons in the world of animal health. Viruses.

[38] Sadeghi S., Tehrani F.R., Tahmasebi S., Shafiee A., Hashemi S.M. (2023). Exosome engineering in cell therapy and drug delivery. Inflammopharmacology.

[39] Wang Z., Mo H., He Z., Chen A., Cheng P. (2022). Extracellular vesicles as an emerging drug delivery system for cancer treatment: Current strategies and recent advances. Biomed. Pharmacother..

[40] Al-Jipouri A., Eritja À., Bozic M. (2023). Unraveling the Multifaceted Roles of Extracellular Vesicles: Insights into Biology, Pharmacology, and Pharmaceutical Applications for Drug Delivery. Int. J. Mol. Sci..

[41] Kwon S., Shin S., Do M., Oh B.H., Song Y., Bui V.D., Lee E.S., Jo D.G., Cho Y.W., Kim D.H. (2021). Engineering approaches for effective therapeutic applications based on extracellular vesicles. J. Control. Release.

[42] Tapparo M., Bruno S., Collino F., Togliatto G., Deregibus M.C., Provero P., Wen S., Quesenberry P.J., Camussi G. (2019). Renal regenerative potential of extracellular vesicles derived from miRNA-engineered mesenchymal stromal cells. Int. J. Mol. Sci..

[43] Kanada M., Bachmann M.H., Hardy J.W., Frimannson D.O., Bronsart L., Wang A., Sylvester M.D., Schmidt T.L., Kaspar R.L., Butte M.J. (2015). Differential fates of biomolecules delivered to target cells via extracellular vesicles. Proc. Natl. Acad. Sci. USA.

[44] Balachandran B., Yuana Y. (2019). Extracellular vesicles-based drug delivery system for cancer treatment. Cogent Med..

[45] van Kouwenhove M., Kedde M., Agami R. (2011). MicroRNA regulation by RNA-binding proteins and its implications for cancer. Nat. Rev. Cancer.

[46] Hagiwara K., Katsuda T., Gailhouste L., Kosaka N., Ochiya T. (2015). Commitment of Annexin A2 in recruitment of microRNAs into extracellular vesicles. FEBS Lett..

[47] Iavello A., Frech V.S.L., Gai C., Deregibus M.C., Quesenberry P.J., Camussi G. (2016). Role of Alix in miRNA packaging during extracellular vesicle biogenesis. Int. J. Mol. Med..

[48] Tapparo M., Pomatto M.A.C., Deregibus M.C., Papadimitriou E., Cavallari C., D’Antico S., Collino F., Camussi G. (2021). Serum Derived Extracellular Vesicles Mediated Delivery of Synthetic miRNAs in Human Endothelial Cells. Front Mol. Biosci..

[49] O’Loughlin A.J., Mäger I., De Jong O.G., Varela M.A., Schiffelers R.M., El Andaloussi S., Wood M.J.A., Vader P. (2017). Functional delivery of lipid-conjugated siRNA by extracellular vesicles. Mol. Ther..

[50] Didiot M.C., Haraszti R.A., Aronin N., Khvorova A. (2018). Loading of extracellular vesicles with hydrophobically modified siRNAs. Methods Mol. Biol..

[51] Roerig J., Schulz-Siegmund M. (2023). Standardization Approaches for Extracellular Vesicle Loading with Oligonucleotides and Biologics. Small.

[52] Baek G., Choi H., Kim Y., Lee H.C., Choi C. (2019). Mesenchymal stem cell-derived extracellular vesicles as therapeutics and as a drug delivery platform. Stem Cells Transl. Med..

[53] Lunavat T.R., Jang S.C., Nilsson L., Park H.T., Repiska G., Lässer C., Nilsson J.A., Gho Y.S., Lötvall J. (2016). RNAi delivery by exosome-mimetic nanovesicles—Implications for targeting c-Myc in cancer. Biomaterials.

[54] de Abreu R.C., Ramos C.V., Becher C., Lino M., Jesus C., da Costa Martins P.A., Martins P.A.T., Moreno M.J., Fernandes H., Ferreira L. (2021). Exogenous loading of miRNAs into small extracellular vesicles. J. Extracell. Vesicles.

[55] Kao C.Y., Papoutsakis E.T. (2018). Engineering human megakaryocytic microparticles for targeted delivery of nucleic acids to hematopoietic stem and progenitor cells. Sci. Adv..

[56] Johnsen K.B., Gudbergsson J.M., Skov M.N., Christiansen G., Gurevich L., Moos T., Duroux M. (2016). Evaluation of electroporation-induced adverse effects on adipose-derived stem cell exosomes. Cytotechnology.

[57] Li W., Szoka F.C. (2007). Lipid-based nanoparticles for nucleic acid delivery. Pharm. Res..

[58] Evers M.J.W., van de Wakker S.I., de Groot E.M., de Jong O.G., Gitz-François J.J.J., Seinen C.S., Sluijter J.P.G., Schiffelers R.M., Vader P. (2022). Functional siRNA Delivery by Extracellular Vesicle-Liposome Hybrid Nanoparticles. Adv. Healthc. Mater..

[59] Pomatto M.A.C., Bussolati B., D’Antico S., Ghiotto S., Tetta C., Brizzi M.F., Camussi G. (2019). Improved loading of plasma-derived extracellular vesicles to encapsulate antitumor miRNAs. Mol. Ther. Methods Clin. Dev..

[60] Goh W.J., Zou S., Lee C.K., Ou Y.H., Wang J.W., Czarny B., Pastorin G. (2018). EXOPLEXs: Chimeric drug delivery platform from the fusion of cell-derived nanovesicles and liposomes. Biomacromolecules.

[61] Shafiei M., Ansari M.N.M., Razak S.I.A., Khan M.U.A. (2021). A comprehensive review on the applications of exosomes and liposomes in regenerative medicine and tissue engineering. Polymers.

[62] Ambrosone A., Barbulova A., Cappetta E., Cillo F., De Palma M., Ruocco M., Pocsfalvi G. (2023). Plant Extracellular Vesicles: Current Landscape and Future Directions. Plants.

[63] Garaeva L., Kamyshinsky R., Kil Y., Varfolomeeva E., Verlov N., Komarova E., Garmay Y., Landa S., Burdakov V., Myasnikov A. (2021). Delivery of functional exogenous proteins by plant-derived vesicles to human cells in vitro. Sci. Rep..

[64] Pomatto M., Gai C., Negro F., Cedrino M., Grange C., Ceccotti E., Togliatto G., Collino F., Tapparo M., Figliolini F. (2021). Differential Therapeutic Effect of Extracellular Vesicles Derived by Bone Marrow and Adipose Mesenchymal Stem Cells on Wound Healing of Diabetic Ulcers and Correlation to Their Cargoes. Int. J. Mol. Sci..

[65] Halperin W., Jensen W.A. (1967). Ultrastructural Changes during Growth and Embryogenesis in Carrot Cell Cultures. J. Ultrastruct. Res..

[66] An Q., Van Bel A.J.E., Hückelhoven R. (2007). Do Plant Cells Secrete Exosomes Derived from Multivesicular Bodies?. Plant Signal. Behav..

[67] Winter V., Hauser M.T. (2006). Exploring the ESCRTing Machinery in Eukaryotes. Trends Plant Sci..

[68] Ruf A., Oberkofler L., Robatzek S., Weiberg A. (2022). Spotlight on Plant RNA-Containing Extracellular Vesicles. Curr. Opin. Plant Biol..

[69] Wang J., Ding Y., Wang J., Hillmer S., Miao Y., Lo S.W., Wang X., Robinson D.G., Jiang L. (2010). EXPO, an Exocyst-Positive Organelle Distinct from Multivesicular Endosomes and Autophagosomes, Mediates Cytosol to Cell Wall Exocytosis in Arabidopsis and Tobacco Cells. Plant Cell.

[70] Ding Y., Wang J., Ho J., Lai C., Hoi V., Chan L., Wang X., Cai Y., Tan X., Bao Y. (2014). Exo70E2 is essential for exocyst subunit recruitment and expo formation in both plants and animals. Mol. Biol. Cell.

[71] Woith E., Guerriero G., Hausman J.F., Renaut J., Leclercq C.C., Weise C., Legay S., Weng A., Melzig M.F. (2021). Plant Extracellular Vesicles and Nanovesicles: Focus on Secondary Metabolites, Proteins and Lipids with Perspectives on Their Potential and Sources. Int. J. Mol. Sci..

[72] Woith E., Fuhrmann G., Melzig M.F. (2019). Extracellular Vesicles—Connecting Kingdoms. Int. J. Mol. Sci..

[73] Movahed N., Cabanillas D.G., Wan J., Vali H., Laliberté J.-F.F., Zheng H. (2019). Turnip Mosaic Virus Components Are Released into the Extracellular Space by Vesicles in Infected Leaves. Plant Physiol..

[74] Rutter B.D., Innes R.W. (2018). Extracellular Vesicles as Key Mediators of Plant-Microbe Interactions. Curr. Opin. Plant Biol..

[75] De La Canal L., Pinedo M. (2018). Extracellular Vesicles: A Missing Component in Plant Cell Wall Remodeling. J. Exp. Bot..

[76] Berin M.C., Shreffler W.G. (2016). Mechanisms underlying induction of tolerance to foods. Immunol. Allergy Clin. N. Am..

[77] Mu J., Zhuang X., Wang Q., Jiang H., Deng Z.B., Wang B., Zhang L., Kakar S., Jun Y., Miller D. (2014). Interspecies communication between plant and mouse gut host cells through edible plant derived exosome-like nanoparticles. Mol. Nutr. Food Res..

[78] Shkryl Y., Tsydeneshieva Z., Degtyarenko A., Yugay Y., Balabanova L., Rusapetova T., Bulgakov V. (2022). Plant Exosomal Vesicles: Perspective Information Nanocarriers in Biomedicine. Appl. Sci..

[79] Alzahrani F.A., Khan M.I., Kameli N., Alsahafi E., Riza Y.M. (2023). Plant-Derived Extracellular Vesicles and Their Exciting Potential as the Future of Next-Generation Drug Delivery. Biomolecules.

[80] Zhuang X., Teng Y., Samykutty A., Mu J., Deng Z., Zhang L., Cao P., Rong Y., Yan J., Miller D. (2016). Grapefruit-derived Nanovectors Delivering Therapeutic miR17 Through an Intranasal Route Inhibit Brain Tumor Progression. Mol. Ther..

[81] Zhang M., Wang X., Han M.K., Collins J.F., Merlin D. (2017). Oral administration of ginger-derived nanolipids loaded with siRNA as a novel approach for efficient siRNA drug delivery to treat ulcerative colitis. Nanomedicine.

[82] Orefice N.S. (2020). Development of New Strategies Using Extracellular Vesicles Loaded with Exogenous Nucleic Acid. Pharmaceutics.

[83] Massaro C., Sgueglia G., Frattolillo V., Baglio S.R., Altucci L., Dell’Aversana C. (2020). Extracellular Vesicle-Based Nucleic Acid Delivery: Current Advances and Future Perspectives in Cancer Therapeutic Strategies. Pharmaceutics.

[84] György B., Szabó T.G., Pásztói M., Pál Z., Misják P., Aradi B., László V., Pállinger E., Pap E., Kittel A. (2011). Membrane vesicles, current state-of-the-art: Emerging role of extracellular vesicles. Cell Mol. Life Sci..

[85] van Niel G., D’Angelo G., Raposo G. (2018). Shedding light on the cell biology of extracellular vesicles. Nat. Rev. Mol. Cell Biol..

[86] Robbins P.D., Morelli A.E. (2014). Regulation of immune responses by extracellular vesicles. Nat. Rev. Immunol..

[87] Wolfers J., Lozier A., Raposo G., Regnault A., Théry C., Masurier C., Flament C., Pouzieux S., Faure F., Tursz T. (2001). Tumor-derived exosomes are a source of shared tumor rejection antigens for CTL cross-priming. Nat. Med..

[88] Zeelenberg I.S., Ostrowski M., Krumeich S., Bobrie A., Jancic C., Boissonnas A., Delcayre A., Le Pecq J.B., Combadière B., Amigorena S. (2008). Targeting tumor antigens to secreted membrane vesicles in vivo induces efficient antitumor immune responses. Cancer Res..

[89] Dutta A. (2021). Exosomes-based cell-free cancer therapy: A novel strategy for targeted therapy. Immunol. Med..

[90] Kashyap D., Panda M., Baral B., Varshney N., Sajitha R., Bhandari V., Parmar H.S., Prasad A., Jha H.C. (2022). Outer Membrane Vesicles: An Emerging Vaccine Platform. Vaccines.

[91] Chronopoulos A., Kalluri R. (2020). Emerging role of bacterial extracellular vesicles in cancer. Oncogene.

[92] Chen S., Lei Q., Zou X., Ma D. (2023). The role and mechanisms of gram-negative bacterial outer membrane vesicles in inflammatory diseases. Front. Immunol..

[93] Holst J., Martin D., Arnold R., Huergo C.C., Oster P., O’Hallahan J., Rosenqvist E. (2009). Properties and Clinical Performance of Vaccines Containing Outer Membrane Vesicles from Neisseria Meningitidis. Vaccine.

[94] van der Pol L., Stork M., van der Ley P. (2015). Outer Membrane Vesicles as Platform Vaccine Technology. Biotechnol. J..

[95] Booth A.M., Fang Y., Fallon J.K., Yang J.M., Hildreth J.E., Gould S.J. (2006). Exosomes and HIV Gag bud from endosome-like domains of the T cell plasma membrane. J. Cell Biol..

[96] Walker J.D., Maier C.L., Pober J.S. (2009). Cytomegalovirus-infected human endothelial cells can stimulate allogeneic CD4+ memory T cells by releasing antigenic exosomes. J. Immunol. Baltim..

[97] Tertel T., Tomić S., Ðokić J., Radojević D., Stevanović D., Ilić N., Giebel B., Kosanović M. (2022). Serum-derived extracellular vesicles: Novel biomarkers reflecting the disease severity of COVID-19 patients. J. Extracell. Vesicles.

[98] Motallebnezhad M., Omraninava M., Esmaeili Gouvarchin Ghaleh H., Jonaidi-Jafari N., Hazrati A., Malekpour K., Bagheri Y., Izadi M., Ahmadi M. (2023). Potential therapeutic applications of extracellular vesicles in the immunopathogenesis of COVID-19. Pathol. Res. Pract..

[99] Pérez P., Astorgano D., Albericio G., Flores S., Sánchez-Cordón P.J., Luczkowiak J., Delgado R., Casasnovas J.M., Esteban M., García-Arriaza J. (2022). Intranasal administration of a single dose of MVA-based vaccine candidates against COVID-19 induced local and systemic immune responses and protects mice from a lethal SARS-CoV-2 infection. Front. Immunol..

[100] Kuate S., Cinatl J., Doerr H.W., Uberla K. (2007). Exosomal vaccines containing the S protein of the SARS coronavirus induce high levels of neutralizing antibodies. Virology.

[101] Cacciottolo M., Nice J.B., Li Y., LeClaire M.J., Twaddle R., Mora C.L., Adachi S.Y., Chin E.R., Young M., Angeles J. (2023). Exosome-Based Multivalent Vaccine: Achieving Potent Immunization, Broadened Reactivity, and Strong T-Cell Responses with Nanograms of Proteins. Microbiol. Spectr..

[102] Cacciottolo M., Li Y., Nice J.B., LeClaire M.J., Twaddle R., Mora C.L., Adachi S.Y., Young M., Angeles J., Elliott K. (2023). Nanograms of SARS-CoV-2 spike protein delivered by exosomes induce potent neutralization of both delta and omicron variants. PLoS ONE.

[103] Ferrantelli F., Chiozzini C., Manfredi F., Giovannelli A., Leone P., Federico M. (2021). Simultaneous CD8^+^ T-Cell Immune Response against SARS-CoV-2 S, M, and N Induced by Endogenously Engineered Extracellular Vesicles in Both Spleen and Lungs. Vaccines.

[104] Ferrantelli F., Chiozzini C., Manfredi F., Leone P., Spada M., Di Virgilio A., Giovannelli A., Sanchez M., Cara A., Michelini Z. (2022). Strong SARS-CoV-2 N-Specific CD8^+^T Immunity Induced by Engineered Extracellular Vesicles Associates with Protection from Lethal Infection in Mice. Viruses.

[105] Jiang L., Driedonks T.A.P., Jong W.S.P., Dhakal S., Bart van den Berg van Saparoea H., Sitaras I., Zhou R., Caputo C., Littlefield K., Lowman M. (2022). A bacterial extracellular vesicle-based intranasal vaccine against SARS-CoV-2 protects against disease and elicits neutralizing antibodies to wild-type and Delta variants. J. Extracell. Vesicles.

[106] Young Chung J., Thone M.N., Davies J.E., Gach J.S., Huw Davies D., Forthal D.N., Kwon Y.J. (2023). Vaccination against SARS-CoV-2 using extracellular blebs derived from spike protein expressing dendritic cells. Cell. Immunol..

[107] Wang Z., Popowski K.D., Zhu D., de Juan Abad B.L., Wang X., Liu M., Lutz H., De Naeyer N., DeMarco C.T., Denny T.N. (2022). Exosomes decorated with a recombinant SARS-CoV-2 receptor-binding domain as an inhalable COVID-19 vaccine. Nat. Biomed. Eng..

[108] Popowski K.D., López de Juan Abad B., George A., Silkstone D., Belcher E., Chung J., Ghodsi A., Lutz H., Davenport J., Flanagan M. (2022). Inhalable exosomes outperform liposomes as mRNA and protein drug carriers to the lung. Extracell. Vesicle.

[109] Pomatto M.A.C., Gai C., Negro F., Massari L., Deregibus M.C., Grange C., De Rosa F.G., Camussi G. (2023). Plant-Derived Extracellular Vesicles as a Delivery Platform for RNA-Based Vaccine: Feasibility Study of an Oral and Intranasal SARS-CoV-2 Vaccine. Pharmaceutics.

[110] Pomatto M.A.C., Gai C., Negro F., Massari L., Deregibus M.C., De Rosa F.G., Camussi G. (2023). Oral Delivery of mRNA Vaccine by Plant-Derived Extracellular Vesicle Carriers. Cells.

[111] Popowski K.D., Moatti A., Scull G., Silkstone D., Lutz H., López de Juan Abad B., George A., Belcher E., Zhu D., Mei X. (2022). Inhalable dry powder mRNA vaccines based on extracellular vesicles. Matter.

[112] Tsai S.J., Atai N.A., Cacciottolo M., Nice J., Salehi A., Guo C., Sedgwick A., Kanagavelu S., Gould S.J. (2021). Exosome-mediated mRNA delivery in vivo is safe and can be used to induce SARS-CoV-2 immunity. J. Biol. Chem..

[113] Chen Z., Xiong M., Tian J., Song D., Duan S., Zhang L. (2024). Encapsulation and assessment of therapeutic cargo in engineered exosomes: A systematic review. J. Nanobiotechnol..

[114] Raghav A., Jeong G.B. (2021). A systematic review on the modifications of extracellular vesicles: A revolutionized tool of nano-biotechnology. J. Nanobiotechnol..

[115] Camussi G., Gai C., Pomatto M.A.C., De Rosa F.G. (2022). Composition Comprising Engineered Plant-Derived Extracellular Vesicles and Use Thereof as a Vaccine.

[116] PLOS BLOGS—Absolutely Maybe: Progress on Intranasal & Oral COVID Vaccines—Plus a US Government Funding Boost. https://absolutelymaybe.plos.org/2023/04/21/progress-on-intranasal-oral-covid-vaccines-plus-a-us-government-funding-boost/?mc_cid=babbbc1bca&mc_eid=6bde7eb6c3#top.

[117] Knisely J.M., Buyon L.E., Mandt R., Farkas R., Balasingam S., Bok K., Buchholz U.J., D’Souza M.P., Gordon J.L., King D.F.L. (2023). Mucosal vaccines for SARS-CoV-2: Scientific gaps and opportunities-workshop report. NPJ Vaccines.

[118] Neutra M.R., Kozlowski P.A. (2006). Mucosal vaccines: The promise and the challenge. Nat. Rev. Immunol..

[119] Corbett K.S., Edwards D.K., Leist S.R., Abiona O.M., Boyoglu-Barnum S., Gillespie R.A., Himansu S., Schäfer A., Ziwawo C.T., DiPiazza A.T. (2020). SARS-CoV-2 mRNA vaccine design enabled by prototype pathogen preparedness. Nature.

[120] Zhang N.N., Li X.F., Deng Y.Q., Zhao H., Huang Y.J., Yang G., Huang W.J., Gao P., Zhou C., Zhang R.R. (2020). A Thermostable mRNA Vaccine against COVID-19. Cell.

[121] Miteva D., Peshevska-Sekulovska M., Snegarova V., Batselova H., Alexandrova R., Velikova T. (2022). Mucosal COVID-19 vaccines: Risks, benefits and control of the pandemic. World J. Virol..

[122] Zafar A., Arshad R., Ur Rehman A., Ahmed N., Akhtar H. (2023). Recent Developments in Oral Delivery of Vaccines Using Nanocarriers. Vaccines.

